# Combination therapeutics of Nilotinib and radiation in acute lymphoblastic leukemia as an effective method against drug-resistance

**DOI:** 10.1371/journal.pcbi.1005482

**Published:** 2017-07-06

**Authors:** Kamran Kaveh, Yutaka Takahashi, Michael A. Farrar, Guy Storme, Marcucci Guido, Jamie Piepenburg, Jackson Penning, Jasmine Foo, Kevin Z. Leder, Susanta K. Hui

**Affiliations:** 1 Program for Evolutionary Dynamics, Harvard University, Cambridge, Massachusetts, United States of America; 2 Department of Radiation Oncology, University of Minnesota, Minneapolis, Minnesota, United States of America; 3 Department of Radiation Oncology, Osaka University, Osaka, Japan; 4 Department of Laboratory Medicine and Pathology, University of Minnesota, Minneapolis, Minnesota, United States of America; 5 Department of Radiotherapy, Universitair Ziekenhuis Brussel, Brussels, Belgium; 6 Department of Hematology and Beckman Research Institute, City of Hope National Medical Center, Duarte, California, United States of America; 7 Department of Mathematics, University of Minnesota, Minneapolis, Minnesota, United States of America; 8 Industrial and Systems Engineering, University of Minnesota, Minneapolis, Minnesota, United States of America; 9 Department of Radiation Oncology, City of Hope National Medical Center and Beckman Research Institute, Duarte, California, United States of America; Institute of Cancer Research, UNITED KINGDOM

## Abstract

Philadelphia chromosome-positive (Ph+) acute lymphoblastic leukemia (ALL) is characterized by a very poor prognosis and a high likelihood of acquired chemo-resistance. Although tyrosine kinase inhibitor (TKI) therapy has improved clinical outcome, most ALL patients relapse following treatment with TKI due to the development of resistance. We developed an *in vitro* model of Nilotinib-resistant Ph+ leukemia cells to investigate whether low dose radiation (LDR) in combination with TKI therapy overcome chemo-resistance. Additionally, we developed a mathematical model, parameterized by cell viability experiments under Nilotinib treatment and LDR, to explain the cellular response to combination therapy. The addition of LDR significantly reduced drug resistance both in vitro and in computational model. Decreased expression level of phosphorylated AKT suggests that the combination treatment plays an important role in overcoming resistance through the AKT pathway. Model-predicted cellular responses to the combined therapy provide good agreement with experimental results. Augmentation of LDR and Nilotinib therapy seems to be beneficial to control Ph+ leukemia resistance and the quantitative model can determine optimal dosing schedule to enhance the effectiveness of the combination therapy.

## Author summary

High likelihood of evolution of resistance to therapy is common in most forms of leukemia. This issue persists for tyrosine kinase inhibitor drug treatments as well as other forms of therapies. In the current work, we suggest a combination therapy where Ph+ acute lymphoblastic leukemic cells are treated with low-dose radiation before chemotherapy (Nilotinib). Our in vitro results of the combined therapy accompanied with a mathematical model shows successful suppression of resistance to Nilotinib. The mathematical model shows a synergistic interaction between Nilotinib and low dose radiation in the chemo dose response function. Beside acute radiation we investigate low dose fractionated therapies with model predicted optimal dosing schedules.

## Introduction

The persistence of chemo-resistant leukemia-initiating cells in Philadelphia-chromosome positive (Ph+) B-cell Acute Lymphoblastic Leukemia (B-ALL) in the bone marrow is a primary mechanism responsible for disease relapse, following treatment, which occurs in the majority of patients. B-ALL is due, in part, to chromosomal translocations (9;22) that result in the generation of a BCR-ABL fusion protein, which fosters the transformation of immature B cells [1]. BCR-ABL+ (i.e., Ph+) leukemia has a poor prognosis; this is particularly true when matched with deletions in Cdkn2a, the gene encoding the tumor suppressor protein ARF, which occurs frequently in B-ALL [2, 3].

A significant breakthrough in the treatment of Ph+ ALL as well as the treatment of chronic myeloid leukemia (CML is associated with p210 isoform, whereas ALL is associated with p190 isoform) was the development of the tyrosine kinase inhibitor (TKI) Imatinib [1]). This drug, and the more potent second generation drugs Dasatinib and Nilotinib, are able to selectively inhibit the BCR-ABL mutant protein and thus significantly reduce Ph+ cell counts [2, 4]. While TKI therapy has long-term efficacy in the treatment of CML, most ALL patients eventually relapse following treatment with TKI due to the development of resistance [5, 6, 7, 8]. Thus a common treatment protocol for ALL patients is TKI therapy until the first remission [9, 10] followed by stem cell transplantation. However, since stem cell transplantation itself carries many risks to patient survival, the ability to extend the efficacy of TKI therapy in Ph+ ALL patients is of great clinical interest. Combination therapy such as Nilotinib with inhibitors of various other pathways (MEK, AKT, and JNK) showed greater reduction in cell viability and lowered risk of resistance [11]. Ionizing radiation has been used for leukemia disease in limited cases, e.g. i) disease involve in the central nervous system (CNS), potential due to ineffective penetration of chemotherapy to CNS [12], (ii) conditioning regimen with high doses of radiation and chemotherapy prior to stem cell transplantation for patients with high risk of relapse [13].

**Fig 1:**
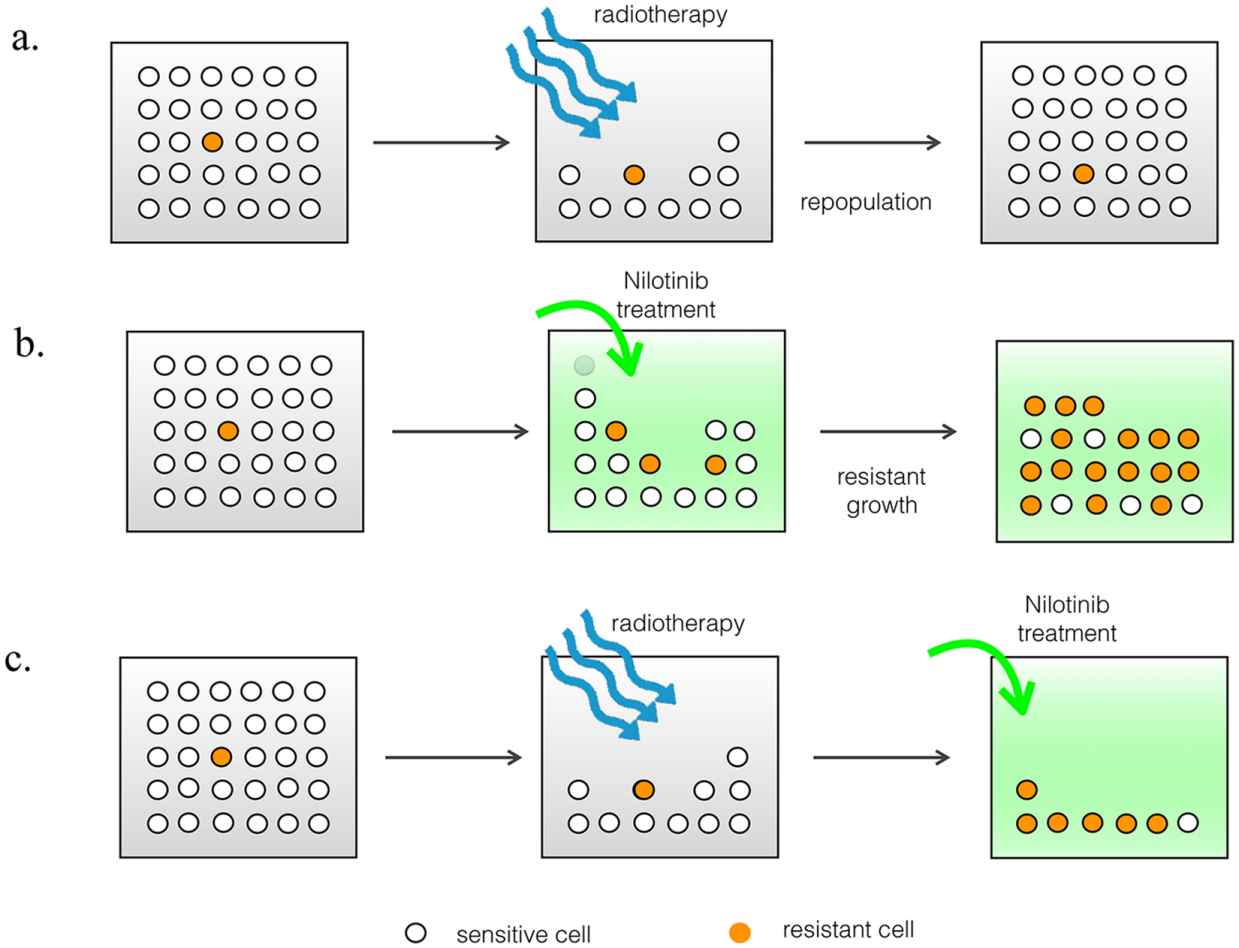
The schematics of sensitive and resistant populations under radiation and/or TKI treatment. a) Soon after acute radiation treatment both cell populations drastically decrease but bounces back through repopulation. (Notice that while a small initial fraction of resistant population is assumed they do not have a growth advantage in the absence of the drug treatment.) b) Upon Nilotinib treatment the sensitive population became disadvantaged and gradually the resistant population outgrows and total population grows back. It does not reach the original population size. c) Combining both therapies both populations are disadvantaged and treatment is much more effective.

Taking advantage of leukemia radiosensitivity and the benefit of low dose radiation (LDR) in preserving bone marrow functions, we investigated whether the combination of Nilotinib and low dose radiation will be more effective treatment for BCR-ABL+ (i.e., Ph+) leukemia over Nilotinib alone. Furthermore, to optimize the effectiveness of this combination treatment, we developed a mathematical model, parameterized via cell viability experiments under Nilotinib treatment and radiation exposure, to predict cellular response to the combination therapy. The optimized mathematical model predicts a synergy between LDR and TKI treatment. We propose a combined Nilotinib dose-response function after LDR that accounts for a possible synergistic interaction between LDR and TKI treatment. Model parameters are obtained from in vitro viability measurements in the absence of TKI (Fig 1(a)), with zero LDR (Fig 1(b)) and combination of LDR and TKI for several radiation doses (Fig 1(c)). The model is validated by precise prediction for the drug-dose responses *and* radiation-dose response to combination LDR and TKI treatment.

It is important to emphasize that our model is focused on the relevance of LDR to prevent small-molecule inhibitor drug resistance. It does not address the efficacy of a successful radiation-drug combination treatment. Answering the question that whether the resistance is pre-existing, selected for or evolves de novo is out the scope of the current work. We do assume a small fraction of pre-existing Nilotinib resistant subtype, as well as possibility to transform into resistant type in the presence of the drug.

## Materials and methods

### Experiments

#### Cell line

Bone marrow was harvested from an Arf-/- (p19-/-) mouse [14]. Red blood cells were lysed and the remaining cells were cultured overnight. Cells were then transduced with a retrovirus containing the BCR-ABL gene, which confers a leukemic phenotype. In that way, B cell acute lymphoblast leukemia cells were obtained and used in this study. Cells were maintained in *α*-MEM supplemented with 10% FBS, 20 mM L-glutamine, penicillin/streptomycin, and *β*-mercaptoethanol.

#### Cell viability assay

**Fig 2:**
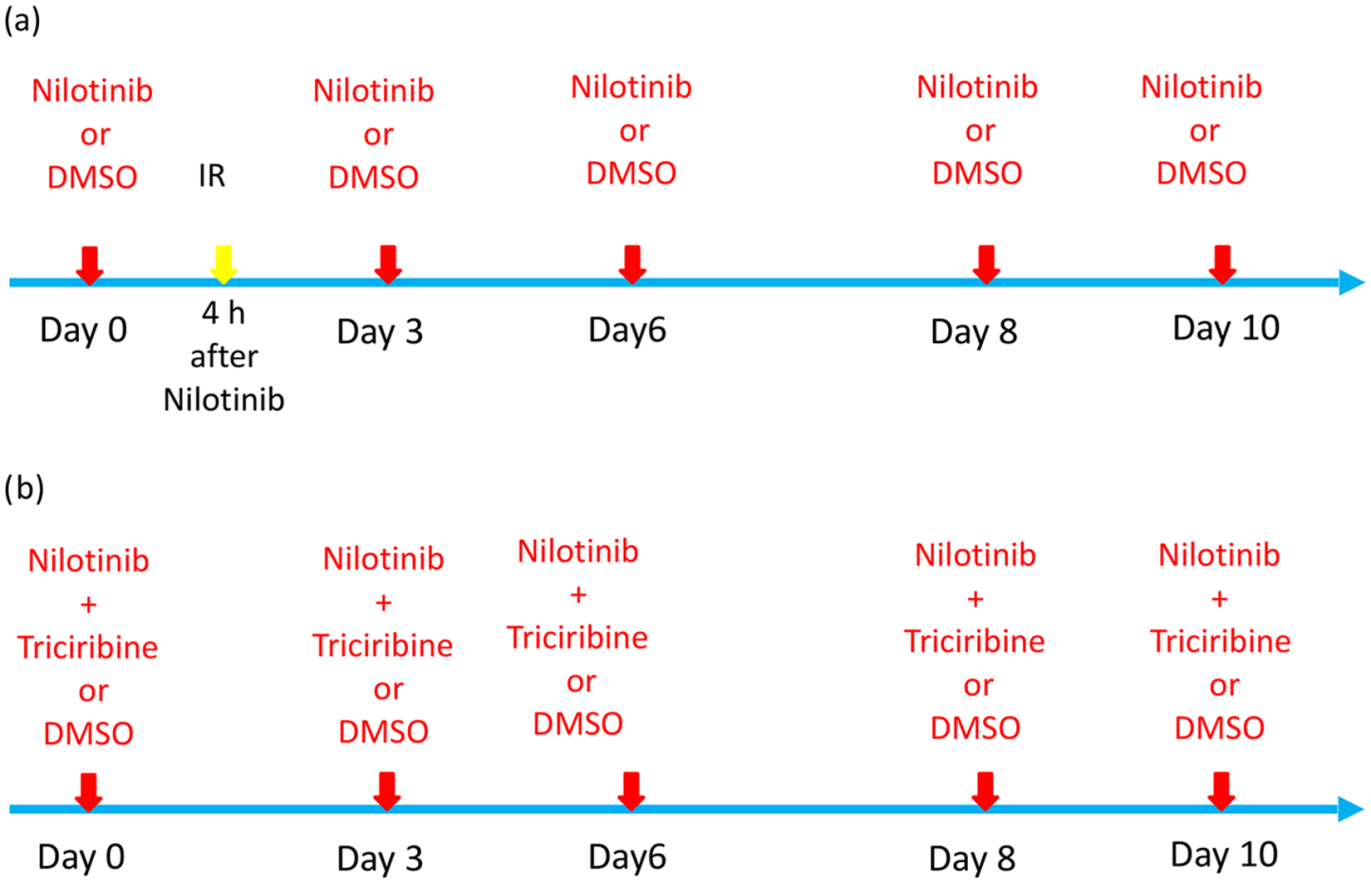
Treatment protocol used in the experiment. (a) Acute radiation dose is administered at day 0 (IR). Doses of Nilotinib is administered every few days to keep the drug concentration in a constant level (day 0, 3, 6, 8, 10). (b) Combined therapy of Nilotinib with Triciribine, an AKT inhibitor, is done for comparison with (a).

We first performed a set of experiments quantifying cell viability as a function of time, for Nilotinib monotherapy, radiation monotherapy, and combined Nilotinib-radiation therapy. For each culture, 5 ×10^5^ cells were plated to each well of a 6-well plate. For the irradiation-only group, cells were irradiated at 2 Gy or 4 Gy on day 0, by 225 kV X-ray beams using X-RAD 320 orthovoltage biological irradiator with a 0.35 mm cupper filter. In the Nilotinib-only group, 18 nM of Nilotinib was added in fresh media at days 0, 3, 6, and 8. For the combination therapy group, 18nM Nilotinib was added to the cells 4 hours before irradiation of 2 Gy or 4 Gy (Fig 2(a)). A boost of Nilotinib with fresh media was administered at days 3, 6, and 8, maintaining Nilotinib on the cells at all times. Cell viability in the combined therapy of 18 nM Nilotinib with Triciribine, an AKT inhibitor, was also conducted as shown in Fig 2(b). The control cells (without treatment) were plated with dimethyl sulfoxide (DMSO) media because DMSO was used to dissolve nilotinib and triciribine drugs. For all treatments, the cell culture medium was changed at days 0, 3, 6, and 8 to maintain the cells. At days 1, 3, 6, 8 and 10, cell viability was assessed by the Trypan blue exclusion assay with a hemocytometer to count the viable and dead cells. Viability was expressed as the percentage of viable cells of the total cell number. All measurements were performed in triplicate. These experiments were utilized to fit the parameters of the mathematical model.

A second set of experiments was performed to determine the dose-response relationship for radiation therapy and Nilotinib monotherapy. For the radiation dose-response assay, under the same plating conditions as described above, cells were administered either 0 or 18 nM Nilotinib for four hours before radiation doses of 0, 1, 2, 4, 6, 8 and 10 Gy on day 0. For the Nilotinib dose-response assay, cells were administered 0, 6, 12.5, or 18 nM Nilotinib on day 0. The viability of each cell population was measured 72 hours after administration of Nilotinib. The results of these experiments were compared with model predictions for model validation purposes.

#### Western blot

To obtain the whole-cell lysates from the cells, the cells were washed in PBS and then lysed in 10 mM Tris-HCl (pH 7.5), 150 mM NaCl, 1 mM EDTA, 1% Triton X-100, 1 mM phenylmethylsulfonyl chloride/isopropanol, and 1 g/ml pepstatin/methanol for 60 min on ice. During incubation, cells were homogenized every 30 minutes using disposable pestles. After centrifugation for 10 min at 14000rpm, the protein was obtained and quantified using BCA Protein Assay kit (ThermoFischer Scientific, Waltham, MA). For each sample, 20ug of total protein were then separated by 10% SDS-PAGE and electroblotted on polyvinylidene difluoride membranes (Bio-Rad, Hercules, CA). After blocking for 1 h in PBS supplement with 5% skim milk, immunodetection of AKT and p-AKT were performed with anti-rabbit monoclonal antibody (1:1000) or antirabbit polyclonal antibody (1:2000;). Anti-rabbit IgG (1:3000) and enhanced chemiluminescence (Amersham Pharmacia Biotech, Aylesbury, United Kingdom) were used for detection.

### Mathematical model

Our computational model is a coupled system of ordinary differential equations representing two populations. A Nilotinib sensitive and a Nilotinib resistant population. The use of ordinary differential equations is very common to describe the population dynamics and emergence of a new trait [15, 16] Logistic terms impose limitation on growth of each population, and an additional term allows for conversion from Nilotinib sensitive to Nilotinib resistant phenotype in the presence of non-zero concentration of the drug. More specifically, we assumed the following for the dynamics of the two subpopulations of the sensitive and resistant phenotypes:

- At the beginning of the experiment, the majority of cells are Nilotinib sensitive. In the absence of the drug, the division rates (and apoptosis rates) of both phenotypes are considered to be the same. Both sensitive and resistant cells are assumed to have similar growth patterns and carrying capacities.
- While we assume a small initial fraction of resistant cells, only in the presence of Nilotinib, resistant cells have a proliferative advantage over Nilotinib sensitive cells. This can be due to depletion of the division rate or increase in the apoptosis rate of the sensitive cells relative to that of the resistant cells.
- In the presence of the drug, sensitive cells can transform at a constant rate into resistant cells. This is due to the fact that random mutations among the sensitive population can give rise to a much more adaptive phenotype for high concentrations of Nilotinib.
- The radiosensitivity of both phenotypes was set to be the same. Furthermore, the effect of TKI treatment on radiosensitivity of both cell types is ignored. Furthermore, Malignant mutations due to low dose ionizing radiation are ignored.

The dynamical model that describes the above mechanisms is detailed in the Supplementary Information (SI). We refer to this model as the proliferation-mutation model.

To identify the response of Ph+ ALL cells to Nilotinib treatment, radiation, and the combination of both therapies, we proposed a simple functional form of this dependence and evaluated the fit against a large set of dose response data from experiment. This simple linear dose-response model can be written as follows:

$$\begin{array}{cc} \left(\text{proliferation}\;\text{rate}\right) & =r_{0}-\left(\text{Nilotinib}\;\text{dose}\right)\times \left(r_{1}+\text{radiation}\;\text{dose}\times r_{2}\right),\\ \left(\text{apoptosis}\;\text{rate}\right) & =d_{0}+\left(\text{Nilotinib}\;\text{dose}\right)\times \left(d_{1}+\text{radiation}\;\text{dose}\times d_{2}\right), \end{array}$$ (1)

where the growth rate coefficients *r*_*i*_ and *d*_*i*_ are constants that need to be identified based on the experimental results. It is common to model dose response functions using a Hill function structure (3- or 4-parameter logistic function). The Hill function imposes a low level of drug efficacy at low doses and a saturation of drug efficacy at high doses. A linear response is most accurate to approximate a Hill function dose-response near IC_50_ does—which is the case here. In the SI section, we show that how the above linear dose-response functions can be derived from a more common Hill -function.

Eq (1) can be rewritten as

$$\begin{array}{ll} r_{\text{S}}= & r_{\text{S},\text{0}}-(r_{\text{S},\text{1}}+r_{\text{S},\text{2}}D)c,\\ r_{\text{R}}= & r_{\text{R},\text{0}}-(r_{\text{R},\text{1}}+r_{\text{R},\text{2}}D)c,\\ d_{\text{S}}= & d_{\text{S},\text{0}}+(d_{\text{S},\text{1}}+d_{\text{S},\text{2}}D)c,\\ d_{\text{R}}= & d_{\text{R},\text{0}}+(d_{\text{R},\text{1}}+d_{\text{R},\text{2}}D)c, \end{array}$$ (2)

where *c* and *D* represent the Nilotinib and radiation doses respectively. The constants *r*_S,0_ and *r*_R,0_ denote the proliferation rates of sensitive and resistant populations in the absence of therapy, and *d*_S,0_ and *d*_R,0_ denote the death rates of sensitive and resistant populations in the absence of therapy. The coefficients *r*_S,1_, *r*_R,1_ represent the dose-response relationship between Nilotinib and proliferation rate of sensitive and resistant cells, respectively. Similarly, the coefficients *d*_S,1_, *d*_R,1_ describe how Nilotinib impacts the death rate of sensitive and resistant cells, respectively. Lastly, the coefficients *r*_S,2_, *r*_R,2_, *d*_S,2_, *d*_R,2_ determine the strength of the radiation-drug interaction on proliferation and death rates sensitive and resistant cells, respectively. These coefficients were fit to experimental data sets studying proliferation and death rates at a variety of Nilotinib and radiation doses. For example, non-negligible positive fitted values of *r*_*S*/*R*,2_ and *d*_*S*/*R*,2_ reveal synergistic interaction between the therapies.

We also incorporated an immediate cell-kill term after each radiation dose in accordance with the standard linear- quadratic model [17, 18, 19, 20]. According to the LQ model the effects of radiation cell kill is given by the survival fraction after the radiation exposure

$$\text{Surviving fraction}=\text{exp}[-\alpha \times \left(\text{radiation dose}\right)-\beta \times \left(\text{radiation dose}{)}^{\text{2}}\right)]$$ (3)

where *α* and *β* are the radio-sensitivity parameters to be determined from the experimental data. In short, *α* represents the rate of cell kill due to single tracks of radiation and *β* represents cell kill due to two independent radiation tracks. The linear quadratic model is widely used due to its excellent agreement with empirical data for a wide range of radiation doses. In SI section we explain how to incorporate the linear quadratic framework for surviving fraction into our mutation-proliferation model.

The above mathematical framework can predict the population fraction (cell viability) at each day given the initial values of Nilotinib and irradiation doses. We fit the model parameters in an iterative fashion. All parameter estimation are obtained by finding optimal parameter set that minimizes the square root distance of solution of Eq. S3 (in SI) with respect to the time series viabilities at days 0, 3, 6, 8, 10. The steps are as follows:

1. Using the cell viability time point data series, in absence of Nilotinib and three radiation doses (0, 2, and 4 Gy), we set the ?control? values of the growth parameters, that is, *r*_*S*,0_ = *r*_*R*,0_, *d*_*S*,0_ = *d*_*R*,0_, *K* (carrying capacity) as well as radio-sensitivity coefficients *α* and *β*.
2. Setting these values, we use viability time series data in the presence of Nilotinib (dose = 18nM) to fit for coefficients *r*_*S*,1_, *r*_*R*,1_, *d*_*R*,2_ and *d*_*S*,2_ as well as *ν*. For this case we use the values *r*_*S*,0_ = *r*_*R*,0_, *d*_*S*,0_ = *d*_*R*,0_, *K* obtained in previous step.
3. The values for *r*_*S*,2_, *r*_*R*,2_, *d*_*R*,2_ and *d*_*S*,2_ are obtained from fitting the viability time series for the combination therapy case with doses 2Gy and 4Gy. For this case we use the values *r*_*S*,0_ = *r*_*R*,0_, *d*_*S*,0_ = *d*_*R*,0_, *K* as well as *r*_*S*,1_, *r*_*R*,1_, *d*_*R*,2_ and *d*_*S*,2_ obtained in previous steps.

## Results

### In vitro experiments

**Fig 3:**
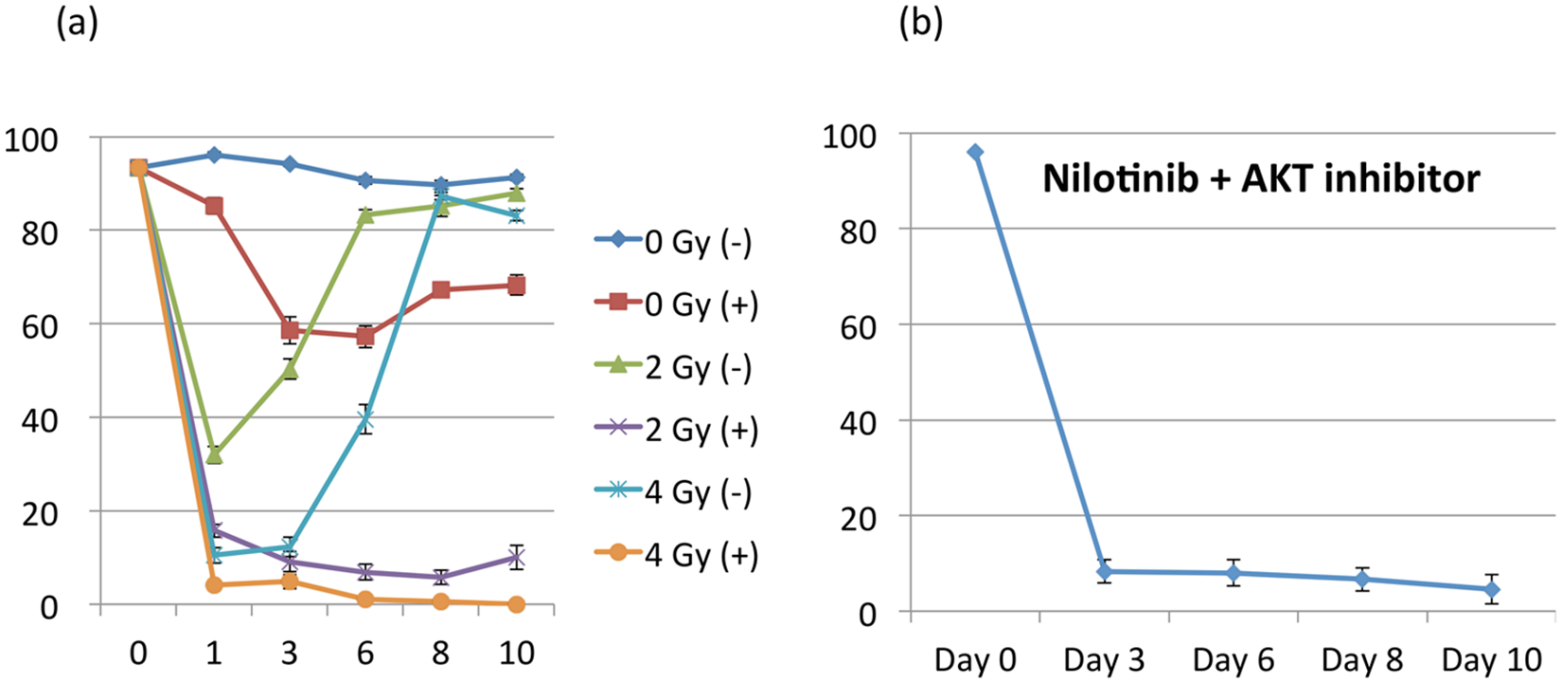
In vitro viability measurements for BCR-ABL+ ALL cell lines in different treatment regimes. (The y-axis is the cell viability in percent and x-axis is time (days).) (a) Cell viability measurements for cell lines treated with low dose radiation and Nilotinib. Plus sign denotes application of Nilotinib (18 nM). b) Similar set up but with AKT inhibitors applied instead of LDR. The results of both low dose radiation and AKT inhibition seems to be the same in preventing the evolution of Nilotinib resistance.

We first measured the time-dependent cell viability of Ph+ ALL cells *in vitro* in response to Nilotinib, radiation, and combination therapy with both Nilotinib and radiation (Fig 3(a)). For the radiation-only arm, we observed an initial large reduction inviability by day 6 for 2 Gy and day 8 for 4 Gy. Experiments using Nilotinib monotherapy showed an incremental reduction in cell viability over the first 6 days to about 57.2%. Subsequently the cell populations began to develop a resistance to the drug and viabilities began to increase. When used in combination, Nilotinib + radiation induced a more effective initial cell killing and the cancer cell population was controlled at very low numbers (under 10% viability) for the duration of the experiment. In other words, there was nearly no cell population recovery, and resistance to Nilotinib was not developed. Under the Nilotinib + 4 Gy treatment arm, the cell population was entirely eliminated. Thus, the combination therapies were able to not only elicit a larger reduction in cell population, but also to maintain control of low cell viabilities without the development of resistance over a longer time period. The use of Triciribine was able to keep the cell viability as low as Nilotinib+ radiation group (Fig 3(b)).

**Fig 4:**
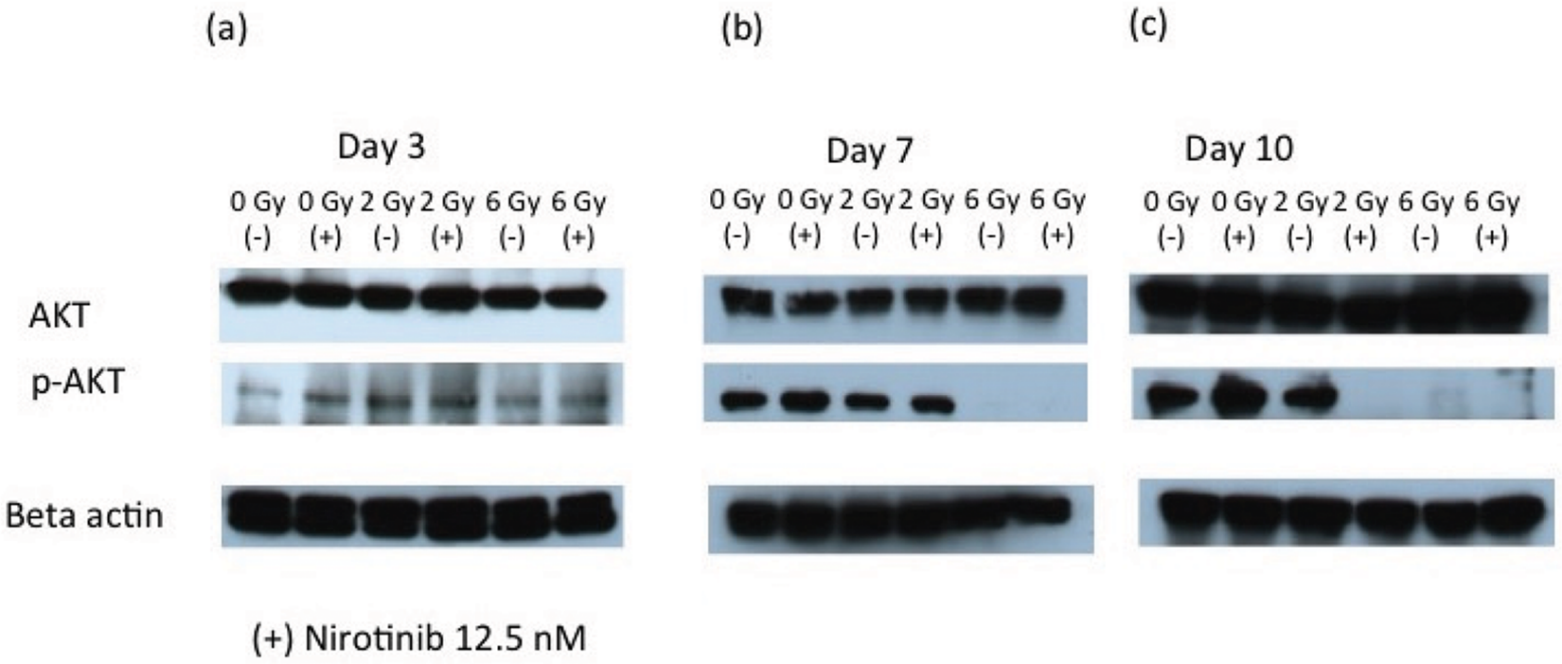
At day 3, AKT was activated by both Nilotinib and radiation. However, after 7 days, p-AKT was down regulated compared to day 3. At day 10, however, Nilotinib without radiation again upregulated p-AKT while combination with radiation kept downregulation. Phospholiation of the AKT may be a critical role in Nilotinib resistant and combination with radiation may killed leukemia cells more efficiently because of p-AKT downreguration.

The radiation dose-response assay revealed an LD50 of 2 Gy in the absence of Nilotinib, and 0.5 Gy in the presence of 18 nM Nilotinib. The Nilotinib dose-response assay revealed an IC_50_ oof 18nM in the absence of radiation. To investigate how the combined therapy provides the synergistic effect, we evaluated one of the key pathways associated with cell proliferation, and chemoresistance. At day 3 after treatment, p-AKT was slightly increased by adding either Nilotinib or radiation (Fig 4(a)). However, the expression level was gradually decreased at large dose of radiation (6 Gy) with or without Nilotinib (Fig 4(b)). Notably, the combined therapy eventually (day 7 and10) decreased the p-AKT even at 2 Gy (Fig 4(c)).

### Fitting the model to experimental data

Using the numerical solutions of the proliferation-mutation model optimized for the best fit with experimental data points, we determined the coefficients in the Nilotinib dose-response function, *r*_*i*_ and *d*_*i*_, as well as the transformation rate (Table 1). The optimized coefficients indicate an increase in fitness of resistant cells relative to that of sensitive cells in the presence of Nilotinib. (Fitness is defined as the difference between division rate and apoptosis rate in the cell population.) Using a numerical sensitivity analysis, we confirmed that these parameter estimates are robust to perturbations in the initial frequency of resistant cells in the population. Since the experiments demonstrated an initially strong sensitivity of the populations to Nilotinib treatment, we set the frequency of resistant cells to be small. We set this to be 0.1% of the total population for the remainder of investigations. The optimized parameters are reported in Table 2 with the carrying capacity set to *K* = 4.2 in all cases. These results indicate the presence of synergistic effects between Nilotinib and radiation. In particular, the radiation tyrosine-kinase inhibitor interaction was strongest in the resistant population. This is not surprising as in the presence of Nilotinib the sensitive population is already highly disadvantaged in terms of growth. The value of transformation rate *ν* in this case is independent of radiation dose and is higher than the respective transformation rate in the Nilotinib-only case suggesting that radiation exposure contributes to the production of new resistant cells.

**Table 1:** Table summary of death rate dose-response coefficients.

*r*_S,0_	*r*_R,0_	*r*_S,1_	*r*_R,1_	*r*_S,2_	*r*_R,2_
2.5369	2.5369	0.0155	0.0	0.0140	0.0
*d*_S,0_	*d*_R,0_	*d*_S,1_	*d*_R,1_	*d*_S,2_	*d*_R,2_
2.0550	2.0550	0.0	0.0	0.0025	0.0114

**Table 2:** Model prediction for proliferation potentials of both sensitive and resistant cells for control, Nilotinib-only and Nilotinib+radiation treatments.

	*r*_s_	*r*_R_	*d*_s_	*d*_R_	*v*
control	2.5369	2.5369	2.0550	2.0550	0.0
nilo-only	2.2579	2.5369	2.0550	2.0550	0.0409
nilo+ 2Gy	1.7539	2.5369	2.1450	2.4654	0.1768
nilo+ 4Gy	1.2499	2.5369	2.2350	2.8758	0.1768

**Fig 5:**
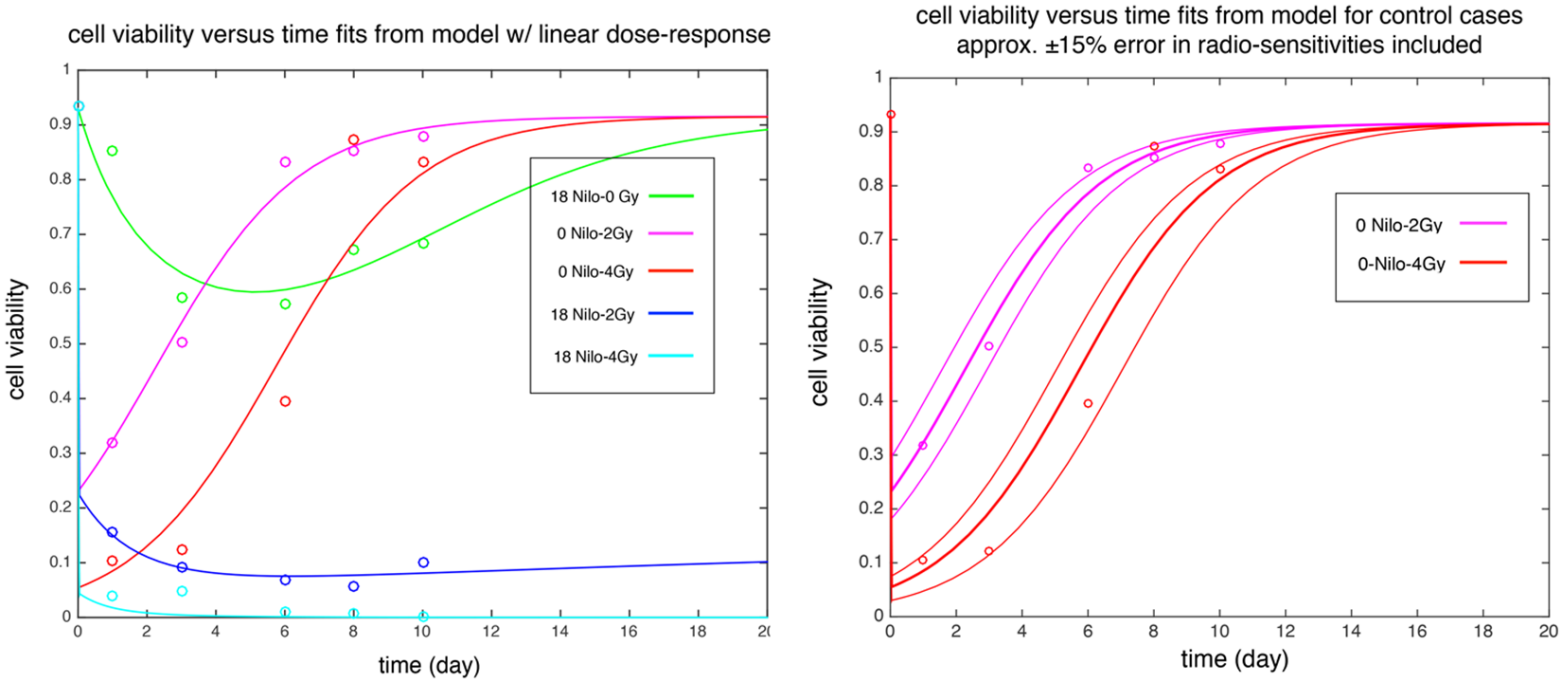
Cell viabilities for control and the treatment cases. a) Time-dependent cell viability *in vitro* under Nilotinib treatment with and without radiation (0-10 days). Solid lines are model fit and circles are experimental results. Red and magenta correspond to 4Gy and 2Gy radiation without Nilotinib (control). b) Time dependent cell viability after 2Gy (magenta) and 4Gy (red) irradiation (day 0). Solid lines show model fit and circles are the in vitro experimental data. Thin lines show model results for varying radiosensitivity parameters by approximately ±15 per cent. The values for parameters are reported in Table 2, *α* = 0.6647, *β* = 0.079 and Δ*α* = ±0.10, Δ*β* = ±0.012.

The results for population fractions (cell viabilities) as a function of time (days) were plotted in Fig 5. The agreement between mathematical model predictions and the in vitro measurements suggests that the proposed radiation-drug interaction term in the linear dose-response is a plausible choice. All the above results of the parameter estimation process are summarized in the Tables 1 and 2.

### Model validation

**Fig 6:**
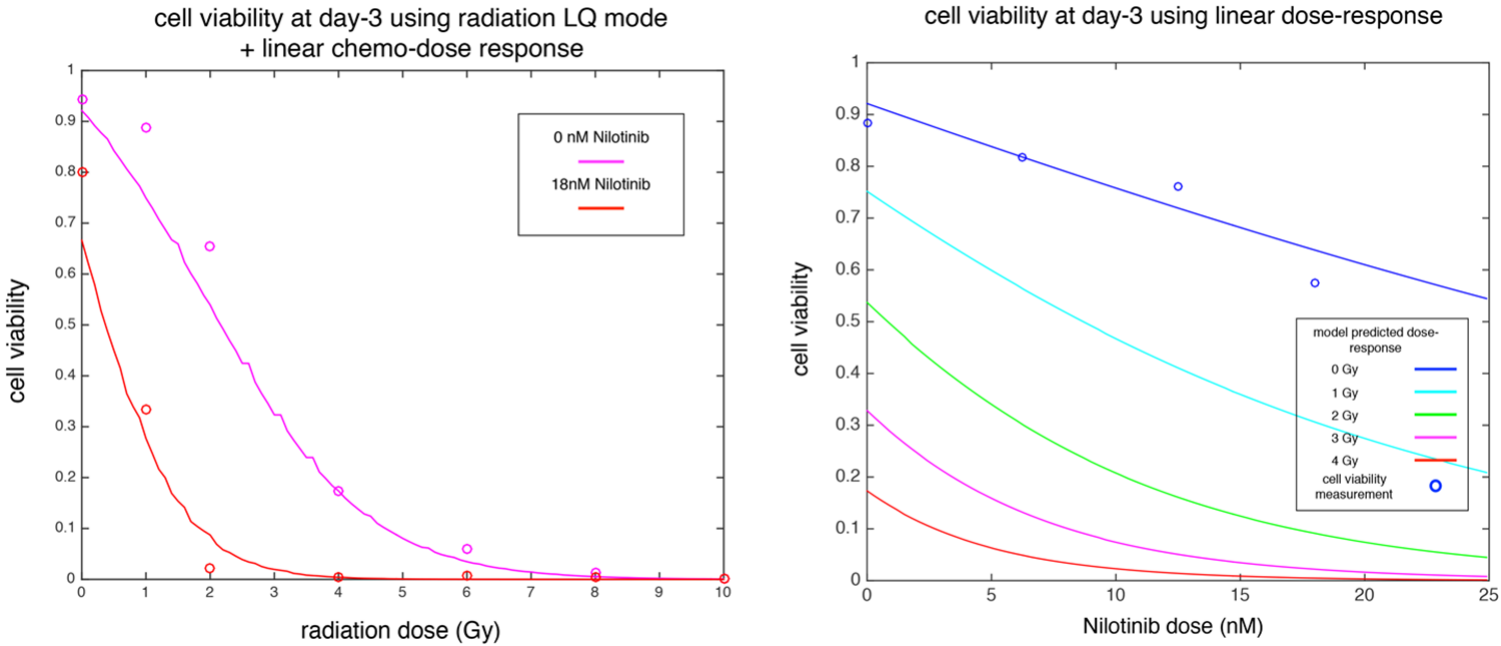
Radiation and Nilotinib dose responses. a) Radiation dose-response curve under no Nilotinib treatment (magenta) and 18 nM Nilotinib (red). Solid lines are model predictions with independently fitted parameters (from Tables 1 and 2). Experimental data shown in circles. Cell viabilities are measured after three days. b) Nilotinib dose-response curves in absence of radiation. Solid lines are model predictions with independently fitted parameters, experimental data shown in circles. Cell viabilities are measured after three days. Other lines represent model predictions for efficacy of Nilotinib treatment combined with various radiation doses of 1, 2, 3 and 4 Gy.

We next tested predictions of the fitted model with an independent set of experimental results. In particular, we compared model predictions to the experimental results of the dose-response assays for Nilotinib (in absence of radiation) and radiation (at 0 and 18 nM Nilotinib). See Fig 6 for these comparisons. As can be seen, the results are in very good agreement. We then used the model to predict the Nilotinib dose-response curve with 1, 2, 3 and 4 Gy radiation. The results are plotted for 0-22nM of Nilotinib. Note that at higher doses we see a discrepancy with the experiment (25nM 0Gy). This is due to the fact that the linear approximation of the Hill dose-response curve only applies to the initial section of the curve and is expected to break down at higher doses.

### Optimization of combination therapies

**Fig 7:**
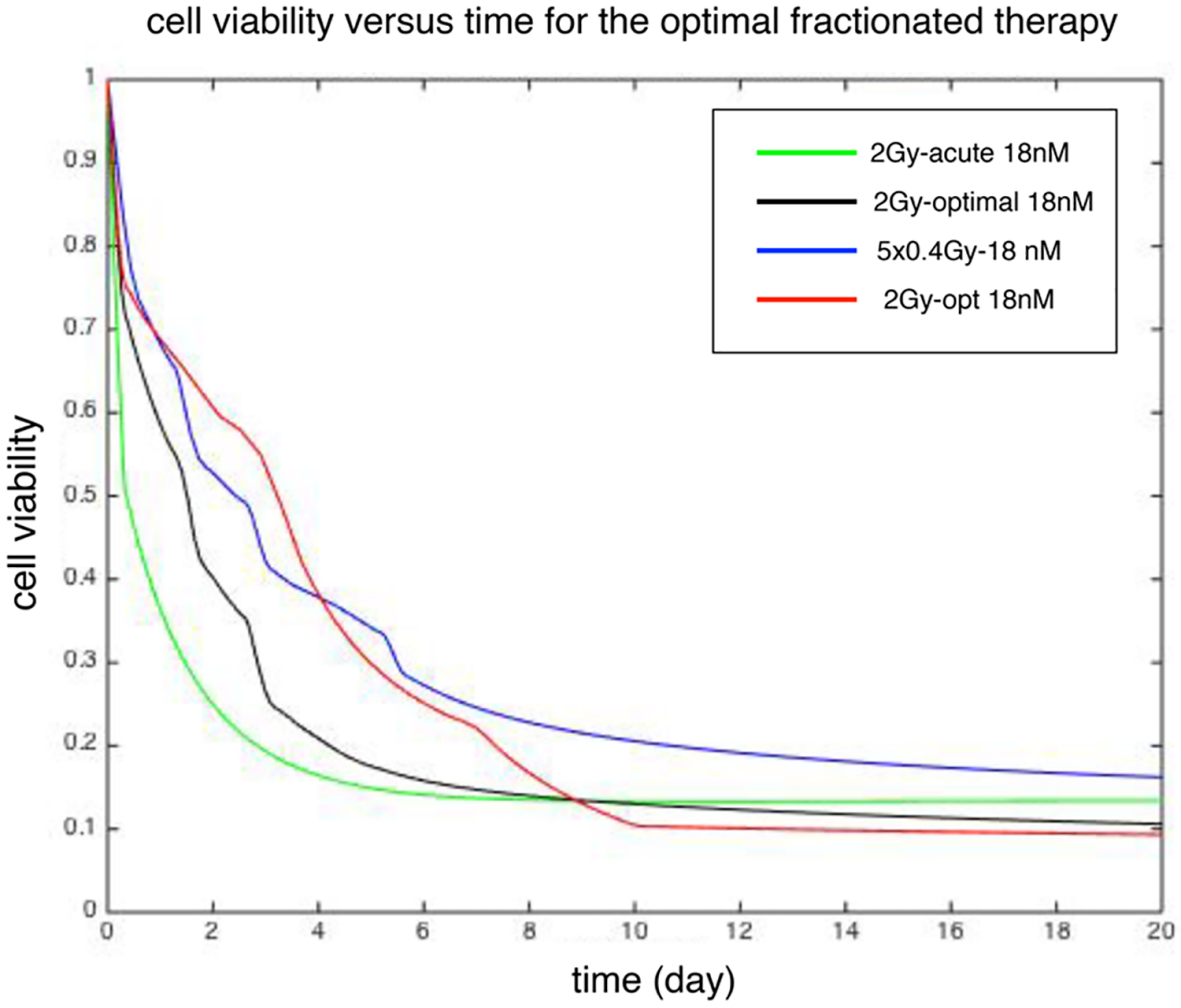
Comparison between three fractionation protocols: 5-fraction with 0.4 Gy per fraction (blue), optimal radiation dose fractions (black) and acute (green) and optimal fractionated radiation + Nilotinib protocols (red). The total cell viabilities is clearly lowest for day 9-10 in the optimal protocol. (See text for details.)

The combination therapy above was shown to reduce tumor cell viability, and through computational techniques an optimal schedule of dosing may be approached. Given the potential toxicity of Nilotinib to normal tissues, we first assumed a standard, constant dose of 18 nM Nilotinib. Next, we considered a five-day radiation treatment protocol, where the summed dose of the protocol is constant. As an example, we use a total dose of 2Gy; which, as can be seen in Fig 5, has room for improvement with the combination therapies and does not irradiate the cells to a viability that is not interesting mathematically (4Gy in Fig 5, for example). The aforementioned result will serve as a comparison for our proposed protocol, which will attempt to minimize the total tumor cell viability at day 10. The control parameters are the radiation doses given at days 0, 1, 2, 3 and 4 denoted by *D*_*i*_(*i* = 1, 2, 3, 4, 5). Placed under the constraint of the total allowable dose, the control parameters were varied by a nonlinear constrained minimization protocol that sought to minimize the tumor cell viability at day 10. Under this minimization protocol, the minimum tumor cell viability was determined by searching the potential dose regimen in the answer space and producing the dosing protocol that best minimized the tumor cell viability at 10 days. This resulted in an optimal dosing schedule of (in Gy) *D*_1_ = 0.9371, *D*_2_ = 0.5139, *D*_3_ = 0.6445, *D*_4_ = 0.045, *D*_4_ = 0.0064, which front-loads the radiation in the first three days. Notice that the negligible value for *D*_4_ indicates that optimal solutions are effectively that of a 4-fraction protocol, with variable doses per fraction. In between any two radiation doses, the cells undergo repopulation. For acute radiation protocols, the linear dose response function predicts a change in proliferation potentials of both sensitive and resistant cells which depends on the total radiation dose at day 0. For a fractionated protocol, we assume the the proliferation potentials of the two cell types between the *k*th and *k*+1th radiation doses depends on total radiation dose until fraction *k*. The model prediction for total cell viabilities versus time for the optimal fractionation protocol is plotted in Fig 7 and compared with a constant 5-fraction radiation protocol (*D*_*i*_ = 0.4) and 2Gy acute radiation treatment (*D*_1_ = 2Gy, *D*_2_ = *D*_3_ = *D*_4_ = *D*_4_ = 0 Gy). At larger time, that is, greater than 10 days, the optimal protocol has the lower cell viability more importantly in a downward trend beyond the 10 day point, whereas the acute treatment begins an upward trend and the constant fractionation protocol does not suppress the cell viability to the levels of the optimal or acute protocols. This suggests the synergistic interactions between the therapies is heightened with larger initial doses of fractionated radiation, and the optimal protocol for long term suppression falls between acute dosing and constant fraction protocols, which results in the front-loading protocol. This dose dependent fractionation may be generalized to various scenarios in the clinical setting as determined by clinical status. Further, the computational model may be extended to consider alternative Nilotinib dosing strategies in combination of the radiation protocol to minimize the cell viability at day 10. Concurrent use of the 5-day optimal fractionation protocol with a varied Nilotinib dosing strategy shows an alternative, though equally efficacious (similar cell viability at 10 day) strategy. This alternative Nilotinib dosing strategy maintains the average daily dose of 18 nM, however, it begins at lower concentration and increases throughout the course of treatment. Specifically, the Nilotinib dose over the first 3 days is 10 nM, followed by 18 nM for 4 days, and finally 26 nM for 3 days, essentially back-loading the Nilotinib onto the irradiated cells. After 10 days, the dose returns to 18 nM daily. This protocol is visualized in Fig 7 as well, where at larger times the new strategy of Nilotinib dosing reduces the cell viability as well as the previous protocol. This is clinically relevant as dose titration is common, if needed, and there is no trade off with treatment efficacy should this dosing schedule be indicated.

## Discussion

Although the clinical outcome of BCR-ABL leukemia has been improved with the advent of TKI therapy, overcoming chemo-resistance has been a major challenge. This study demonstrated that low dose irradiation combined with Nilotinib provided enhanced and prolonged efficacy for leukemic cells in vitro. A companion theoretical model provided good agreement with experimental results with an opportunity for further optimization to enhance treatment efficacy. We explored here the possibility of using low dose radiation as a first line therapy in combination with chemotherapy to enhance the effectiveness controlling BCR-ABL leukemia. There are mainly two reasons for such a combination therapy approach: a) advent of image guided targeted radiation allow more focused dose delivery [21, 22, 23] b) known radiosensitivity of leukemia but not known if low dose radiation with chemotherapy could be an effective alternative to control over the TKI drug resistance. We found that combining low dose of radiation with a commonly used TKI drug could not only substantially reduce cell population, but also maintain low cell viabilities without the development of resistance over a longer time period. To elucidate the mechanism for which low dose radiation provide beneficial effect, we investigated the role of AKT pathway using western blot analysis (Fig 4). Our results revealed that high dose radiation (i.e. 6 Gy) reduced the phosphorylation of AKT. Interestingly, even low dose radiation was adequate to inhibit the phosphorylation of AKT when Nilotinib was also used. To support of this, the cell viability of chemo-resistant cells (with increased p-AKT) was significantly reduced when AKT inhibitor (Triciribine) was used in combination with Nilotinib. These results suggest that radiation can play roles not only in a conditioning regimen before stem cell transplant but also an alternative for an AKT inhibitor. In other words, low dose radiation therapy could be an alternative treatment option for AKT inhibitor in ALL patients. To our best knowledge, this is the first report that demonstrates the promising combined efficacy of Nilotinib and radiation for ALL, with minimum damage to vital organs. Because of low dose with limited toxicity, it can be delivered to the whole body. Since our study was performed in normal cell culture, however, in vivo study is required to take into consideration of microenvironmental factors.

We also constructed a dynamical model to explain the observations and to predict response to additional combination therapy schedules. To tease apart the responses of Ph+ ALL cells to Nilotinib, radiation, and the combination of both therapies, we proposed a simple functional form of the combination dose-response relationship and evaluated the fit against a large set of dose response data. In particular, we proposed a simple Nilotinib dose-response function in which radiation dose may alter the strength of the Nilotinib response in a dose-dependent fashion. Parameter-fitting revealed an optimal parameter set that showed very good agreement with experimental results. Analysis of this optimal parameter set revealed dose-dependent synergistic effects between Nilotinib and radiation response. In particular the radiation tyrosine-kinase inhibitor interaction is strongest in the resistant subpopulation of cells. Our analysis also demonstrated that the model predictions are robust to variation in the initial frequency of resistant cells. We compared fitted model predictions with an independently generated second set of experimental data and found good agreement.

We next utilized the validated model to investigate optimal combination strategies for Nilotinib and radiation in Ph+ ALL. As a simple test, we assumed a standard 18 nM Nilotinib dose and investigated strategies allowing up to 2 Gy over the course of a 5-day treatment. We determined that the optimal therapeutic schedule, given these constraints, spread most of the radiation dose over the first three days. Thus, a ‘sweetspot’ exists between acute radiation protocols and constant treatment protocols. These results suggest a promising direction for investigation of new treatment strategies in Ph+ ALL and for providing an optimal treatment regimen. However, we note that all experiments (and thus parametrization of the model) was done in vitro. Further in vivo studies are needed to determine treatment schedules for the clinic with more accuracy. To summarize, augmentation of LDR-Nilotinib therapy may be beneficial to control Ph+ve leukemia resistance and the model can determine optimal dosing schedule to enhance the effectiveness of the combination therapy.

## Supporting information

S1 Text Extended sections on theory and materials and methods. (PDF)

## References

[1] Deininger M, Buchdunger E, Druker BJ. The development of imatinib as a therapeutic agent for chronic myeloid leukemia. Blood. 2005;105(7):2640–2653. 10.1182/blood-2004-08-3097 15618470

[2] Talpaz M, Shah NP, Kantarjian H, Donato N, Nicoll J, Paquette R, et al Dasatinib in imatinib-resistant Philadelphia chromosome–positive leukemias. New England Journal of Medicine. 2006;354(24):2531–2541. 10.1056/NEJMoa055229 16775234

[3] Shimoni A, Volchek Y, Koren-Michowitz M, Varda-Bloom N, Somech R, Shem-Tov N, et al Phase 1/2 study of nilotinib prophylaxis after allogeneic stem cell transplantation in patients with advanced chronic myeloid leukemia or Philadelphia chromosome–positive acute lymphoblastic leukemia. Cancer. 2015;121(6):863–871. 10.1002/cncr.29141 25387866

[4] Kantarjian H, Giles F, Wunderle L, Bhalla K, O’Brien S, Wassmann B, et al Nilotinib in imatinib-resistant CML and Philadelphia chromosome–positive ALL. New England Journal of Medicine. 2006;354(24):2542–2551. 10.1056/NEJMoa055104 16775235

[5] Gaynon PS. Childhood acute lymphoblastic leukaemia and relapse. British journal of haematology. 2005;131(5):579–587. 10.1111/j.1365-2141.2005.05773.x 16351633

[6] Pui CH, Evans WE. Treatment of acute lymphoblastic leukemia. New England Journal of Medicine. 2006;354(2):166–178. 10.1056/NEJMra052603 16407512

[7] Den Boer ML, van Slegtenhorst M, De Menezes RX, Cheok MH, Buijs-Gladdines JG, Peters ST, et al A subtype of childhood acute lymphoblastic leukaemia with poor treatment outcome: a genome-wide classification study. The lancet oncology. 2009;10(2):125–134. 10.1016/S1470-2045(08)70339-5 19138562 PMC2707020

[8] Yang K, Fu Lw. Mechanisms of resistance to BCR–ABL TKIs and the therapeutic strategies: A review. Critical reviews in oncology/hematology. 2015;93(3):277–292. 10.1016/j.critrevonc.2014.11.001 25500000

[9] Ottmann O. Management of Philadelphia chromosome-positive acute lymphoblastic leukemia. Leukemia supplements. 2012;1:S7–S9. 10.1038/leusup.2012.7 27175253 PMC4851212

[10] Ottmann OG, Pfeifer H. Management of Philadelphia chromosome–positive acute lymphoblastic leukemia (Ph+ ALL). ASH Education Program Book. 2009;2009(1):371–381. 10.1182/asheducation-2009.1.371 20008223

[11] Feldhahn N, Arutyunyan A, Stoddart S, Zhang B, Schmidhuber S, Yi SJ, et al Environment-mediated drug resistance in Bcr/Abl-positive acute lymphoblastic leukemia. Oncoimmunology. 2012;1(5):618–629. 10.4161/onci.20249 22934254 PMC3429566

[12] Kwon JH, Koh Yi, Yoon Ss, Park S, Kim I. Clinical outcome and efficacy of current anti-leukemic therapy for leptomeningeal involvement in acute myeloid leukemia. International journal of hematology. 2016;104(5):574–581. 10.1007/s12185-016-2063-6 27431487

[13] Wong JY, Forman S, Somlo G, Rosenthal J, Liu A, Schultheiss T, et al Dose escalation of total marrow irradiation with concurrent chemotherapy in patients with advanced acute leukemia undergoing allogeneic hematopoietic cell transplantation. International Journal of Radiation Oncology* Biology* Physics. 2013;85(1):148–156. 10.1016/j.ijrobp.2012.03.033 PMC4312108 22592050

[14] Kamijo T, Zindy F, Roussel MF, Quelle DE, Downing JR, Ashmun RA, et al Tumor suppression at the mouse INK4a locus mediated by the alternative reading frame product p19 ARF. Cell. 1997;91(5):649–659. 10.1016/S0092-8674(00)80452-3 9393858

[15] Komarova NL, Wodarz D. Targeted cancer treatment in silico. Springer; 2014.

[16] Panetta JC. A mathematical model of drug resistance: heterogeneous tumors. Mathematical biosciences. 1998;147(1):41–61. 10.1016/S0025-5564(97)00080-1 9401351

[17] Chadwick K, Leenhouts H. A molecular theory of cell survival. Physics in medicine and biology. 1973;18(1):78 10.1088/0031-9155/18/1/007 4803965

[18] Dale RG, Jones B, et al Radiobiological modelling in radiation oncology. British Institute of Radiology; 2007.

[19] Hall EJ, Giaccia AJ. Radiobiology for the Radiologist. Lippincott Williams and Wilkins; 2006.

[20] Brenner DJ, Hlatky LR, Hahnfeldt PJ, Hall EJ, Sachs RK. A convenient extension of the linear-quadratic model to include redistribution and reoxygenation. International Journal of Radiation Oncology* Biology* Physics. 1995;32(2):379–390. 10.1016/0360-3016(95)00544-9 7751180

[21] Hui SK, Kapatoes J, Fowler J, Henderson D, Olivera G, Manon RR, et al Feasibility study of helical tomotherapy for total body or total marrow irradiationan. Medical physics. 2005;32(10):3214–3224. 10.1118/1.2044428 16279075

[22] Magome T, Froelich J, Takahashi Yea. Evaluation of functional marrow irradiation based on skeletal marrow composition obtained using dual-energy CT. international Journal of Radiation Oncology. 2016;. 10.1016/j.ijrobp.2016.06.2459 PMC5081224 27681765

[23] Rosenthal J, Wong J, Stein A, Qian D, Hitt D, Naeem H, et al Phase 1/2 trial of total marrow and lymph node irradiation to augment reduced-intensity transplantation for advanced hematologic malignancies. Blood. 2011;117(1):309–315. 10.1182/blood-2010-06-288357 20876852 PMC3037752

