# Supplementary material for: Combination therapeutics of Nilotinib and radiation in acute lymphoblastic leukemia as an effective method against drug-resistance
Source: PLoS Comput Biol. 2017 Jul 6;13(7):e1005482. doi: 10.1371/journal.pcbi.1005482 (PMC5500007; doi:10.1371/journal.pcbi.1005482)
Supplement: S1 Text — (PDF) [file pcbi.1005482.s001.pdf]

Supporting Information

0.1 Derivation of the linear dose-response function

It is common to use a Hill function type for dose response. For example the death rate, d, of one of the two subpopulation (either resistant or sensitive) with drug dose c applied, is written as

$$d(c) = \hat{d}_0 + \frac{\hat{d}_\infty - \hat{d}_0}{1 + \left(\frac{\mathrm{IC}_{50}}{c}\right)^\gamma} \tag{1}$$

The death rate d_0 represents the control value in the absence of the drug, d_∞ is the death rate in very high doses, γ is the Hill exponent and IC_{50} is the drug concentration where the inhibition factor (death rate is this case) is reduced by a one-half. The above function can be taylor expanded for $c = \mathrm{IC}_{50}$. The results to linear order in $c - IC_{50}$ are

$$d(c) = \frac{2+\gamma}{4}\big(\hat{d}_0 + \hat{d}_\infty\big) + \frac{1}{4}\frac{\hat{d}_0 - \hat{d}_\infty}{\mathrm{IC}_{50}} \cdot c + \mathcal{O}\Big((c - \mathrm{IC}_{50})^2\Big) \tag{2}$$

This is the linearized dose-response function we used where $d_0 = \frac{2+\gamma}{4}\big(\hat{d}_0 + \hat{d}_\infty\big)$ and $d_1 = \hat{d}_\infty + (1/4)(\hat{d}_0 - \hat{d}_\infty)/\mathrm{IC}_{50}$. To include the radiation-drug interaction we propose to have the $c \to c \cdot (1 + d_2 D)$. The higher order terms, i.e. $(c - \mathrm{IC}_{50})^2$ becomes more significant for very low or very high drug doses and are responsible for discrepancy with the measurements observed in dose-response in those regimes.

0.2 Mutation-proliferation model

In this section we present a simple model of mutation-proliferation that describes the dynamics of sensitive and resistant populations in the system. We denote the abundance of sensitive cells with x_S and resistants with x_R (normalized to the initial population size). Also the division rate of sensitive cells are denoted by r_S and death rate with d_S. Similarly, resistant phenotype divide and die with rates r_R and d_R. The total population can reach a maximum viability indicated by K. We assume a constant mutation or transformation rate, ν, where sensitive cells can transform into resistant. We write the killing induced by radiation treatment separately. From the linear-quadratic formula (see next section) this can be written proportional to dose delivery rate dD/dt. Where $D(t)$ is total dose delivered up to time t. A schematic of the possible proliferation and death events, and their corresponding rates of occurrence, are depicted in Fig. A.

This dynamics can be captured in the following system of ordinary differential equations,

$$\begin{aligned} \frac{\mathrm{d}x_\mathrm{S}}{\mathrm{d}t} &= r_\mathrm{S} x_\mathrm{S}\Big(1 - \frac{x_\mathrm{S} + x_\mathrm{R}}{K}\Big) - d_\mathrm{S} x_\mathrm{S} - \frac{\mathrm{d}D}{\mathrm{d}t}\big(\alpha + 2\beta D(t)\big) x_\mathrm{S} - \nu x_\mathrm{S}, \\ \frac{\mathrm{d}x_\mathrm{R}}{\mathrm{d}t} &= r_\mathrm{R} x_\mathrm{R}\Big(1 - \frac{x_\mathrm{S} + x_\mathrm{R}}{K}\Big) - d_\mathrm{R} x_\mathrm{R} - \frac{\mathrm{d}D}{\mathrm{d}t}\big(\alpha + 2\beta D(t)\big) x_\mathrm{R} + \nu x_\mathrm{S}. \end{aligned} \tag{3}$$

As discussed in the text we have assumed that radio-sensitivity parameters α and β are the same for both phenotypes. We assume both proliferation rates and death rates

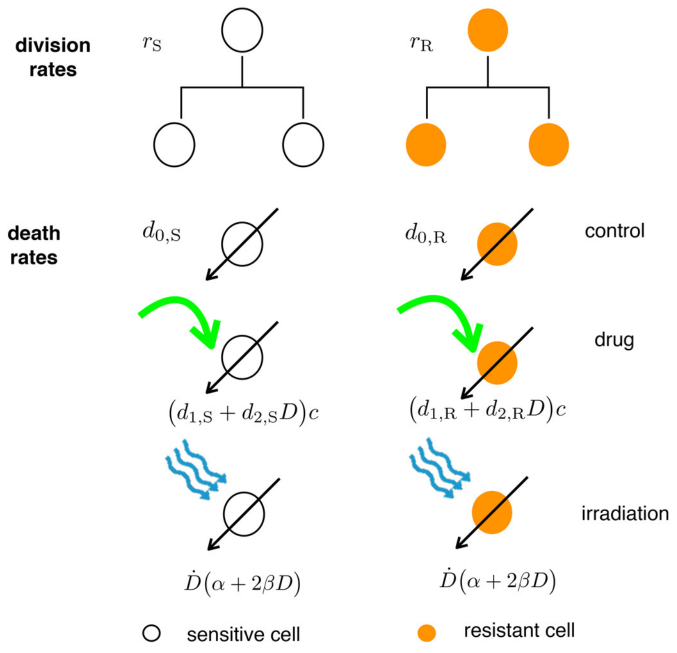

Fig A. A schematic of possible proliferation and death events and corresponding rates in our model. For brevity the effect of Nilotinib and radiation on death rates are shown. In principle Nilotinib affects division/proliferation rates as well.

are affected by Nilotinib concentration. As discussed in the main text we use a linear regression dose response function given by Eq.2. The continuous-time representation of the linear-quadratic formula that is used here is previously been use in the literature of radiation-oncology (see for example [1]).

As discussed in the text, for the control group the transformation rate, ν, is zero and proliferation and death rates of both populations are assumed to be equal. In this case the solutions of the proliferation-mutation model are straightforward. Denoting total abundance of cells by $x(t)$ and initial cell viability as $x_0 = \exp(-\alpha D - \beta D^2)$ we have

$$x(t) = \frac{e^{-\alpha D-\beta D^2} e^{(r-d)t}(r-d)}{e^{-\alpha D-\beta D^2}(e^{(r-d)t}-1)r + r - d} \tag{4}$$

In fact we can use the above results to analytically approximate the values of radio-sensitivity coefficients. (See next section of SI)

0.3 Continuous-time linear-quadratic formula

Effect of ionizing radiation on malignant/or normal cells is two fold. Single-strand or double-strand breaks of DNA causes some of the cells to undergo apoptosis. Also with a much smaller probability ionizing radiation can cause mutations. In the following we ignore the mutation mechanism. The survival fraction, S, of cells that have been exposed to a radiation dose of D can be expressed by linear-quadratic formula,

$$S = e^{-\alpha D-\beta D^2}, \tag{5}$$

where α and β are radio-sensitivity parameters. We can express this in a continuous time format as well. In the absence of the cell proliferations we write the dynamics of total population as

$$\frac{\mathrm{d}x}{\mathrm{d}t} = -x(t)\big(\alpha + 2\beta D(t)\big)\frac{\mathrm{d}D}{\mathrm{d}t} \tag{6}$$

we can change variable from time t to dose delivered until time t, $D(t)$. The solutions for $x(t)$ as a function of dose are,

$$x(t) = x_0 e^{-\alpha D(t) - \beta D^2(t)} \tag{7}$$

where x_0 is the population before treatment. The survival fraction $x(t)/x_0$ is the thus given by well-known linear-quadratic formulae.

We can use the numerical values of the survival fraction in in vitro experiments to estimates the radio-sensitivity coefficients α and β. If the cell viabilities are measured right after irradiation for two separate administered doses D_1 and D_2 we have

$$\begin{aligned} -\ln(S_1) &= \alpha D_1 + \beta D_1^2 \\ -\ln(S_2) &= \alpha D_2 + \beta D_2^2 \end{aligned} \tag{8}$$

which gives

$$\begin{aligned} \alpha &= \frac{D_2^2 \ln S_1 - D_1^2 \ln S_2}{D_2 D_1^2 - D_1 D_2^2} \\ \beta &= \frac{D_2 \ln S_1 - D_1 \ln S_2}{D_2 D_1^2 - D_1 D_2^2} \end{aligned} \tag{9}$$

If the cell viabilities are measured after item t, which gives time for a partial repopulation of the cells we need to take repopulation dynamics into account. Denoting death and birth rates as r and d and S_1, S_2 as cell viabilities measured at time t after irradiation then radio-sensitivities are

$$\begin{aligned} \alpha &= \frac{D_2^2 \ln\left(\frac{S_1(d-r)}{(S_1-1)r+d)e^{-(d-r)t} - S_1 r}\right) - D_1^2 \ln\left(\frac{S_2(d-r)}{(S_2-1)r+d)e^{-(d-r)t} - S_2 r}\right)}{D_1 D_2 (D_1 - D_2)} \\ \beta &= \frac{-D_2 \ln\left(\frac{S_1(d-r)}{(S_1-1)r+d)e^{-(d-r)t} - S_1 r}\right) + D_1 \ln\left(\frac{S_2(d-r)}{(S_2-1)r+d)e^{-(d-r)t} - S_2 r}\right)}{D_1 D_2 (D_1 - D_2)} \end{aligned} \tag{10}$$

References

1. Kaveh K, Manem VS, Kohandel M, Sivaloganathan S. Modeling age-dependent radiation-induced second cancer risks and estimation of mutation rate: an evolutionary approach. Radiation and environmental biophysics. 2015;54(1):25–36.
